# Visualization and exploratory analysis of epidemiologic data using a novel space time information system

**DOI:** 10.1186/1476-072X-3-26

**Published:** 2004-11-08

**Authors:** Gillian A AvRuskin, Geoffrey M Jacquez, Jaymie R Meliker, Melissa J Slotnick, Andrew M Kaufmann, Jerome O Nriagu

**Affiliations:** 1 BioMedware Inc., 516 N. State St., Ann Arbor, MI 48104, USA; 2 Department of Environmental Health Sciences, School of Public Health, University of Michigan, Ann Arbor, MI 48109–2029, USA

## Abstract

**Background:**

Recent years have seen an expansion in the use of Geographic Information Systems (GIS) in environmental health research. In this field GIS can be used to detect disease clustering, to analyze access to hospital emergency care, to predict environmental outbreaks, and to estimate exposure to toxic compounds. Despite these advances the inability of GIS to properly handle temporal information is increasingly recognised as a significant constraint. The effective representation and visualization of both spatial and temporal dimensions therefore is expected to significantly enhance our ability to undertake environmental health research using time-referenced geospatial data. Especially for diseases with long latency periods (such as cancer) the ability to represent, quantify and model individual exposure through time is a critical component of risk estimation. In response to this need a STIS – a Space Time Information System has been developed to visualize and analyze objects simultaneously through space and time.

**Results:**

In this paper we present a "first use" of a STIS in a case-control study of the relationship between arsenic exposure and bladder cancer in south eastern Michigan. Individual arsenic exposure is reconstructed by incorporating spatiotemporal data including residential mobility and drinking water habits. The unique contribution of the STIS is its ability to visualize and analyze residential histories over different temporal scales. Participant information is viewed and statistically analyzed using dynamic views in which values of an attribute change through time. These views include tables, graphs (such as histograms and scatterplots), and maps. In addition, these views can be linked and synchronized for complex data exploration using cartographic brushing, statistical brushing, and animation.

**Conclusion:**

The STIS provides new and powerful ways to visualize and analyze how individual exposure and associated environmental variables change through time. We expect to see innovative space-time methods being utilized in future environmental health research now that the successful "first use" of a STIS in exposure reconstruction has been accomplished.

## Background

Geographic Information Systems are beneficial tools in modelling static representations of reality; however they fall short in their ability to handle time. The ability to store, visualize, and analyze both the temporal and spatial dimension of data continues to be a challenging task. Over the past decade, there have been several attempts to include time enabled capabilities into GIS. [1] and [2] proposed amendment vectors to extend the vector data model to the time dimension, while others enhanced the grid data model to represent snap-shots of raster data at different time intervals [3]. Although temporal extensions exist, e.g. [2] commercial GIS packages do not properly support temporal aspects of spatial data [4].

The importance of GIS for medical research and epidemiology has long been recognized [5-7], and GIS is frequently used for retrospective exposure reconstruction [8-10]. However the application of GIS to risk and exposure assessment has historically focused on the hazard as the object of interest – such as the locations of contaminated industrial sites with high concentrations of carcinogens – instead of the individual [3]. More recently exposure assessment using GIS has targeted individuals in their present homes, but relatively little attention has been placed on individual exposure reconstruction involving residential histories and past activities. This in large part is due to the poor ability of current GISs to handle multitemporal geographic information and the movement of individuals within the context of putative exposure sources whose locations and output change through time. Consequently, there have been few attempts to expand on the 'static map' to provide a more accurate view of exposure.

The ability to effectively represent, query, and model the temporal dimension is expected to significantly enhance researchers' abilities to undertake environmental health research with georeferenced data. Studying an individual's exposure over time is a key factor in determining risk, particularly for diseases with long latency periods such as cancer [3], because individual exposure to environmental contaminants (eg carcinogens) can change as people move through space over time. Exposure assessment characterizes the concentration of potential toxins, as well as the frequency and duration of contacts between individuals and those toxins. Therefore, accurate exposure assessment requires estimation of variation in contaminant concentration as well as changes in geographic proximity to contaminant sources over time. This requires models that can account for residential histories and how residential location influences ambient contaminant concentrations as well as exposure opportunities.

In this research we applied a STIS to visualize and analyze data from a bladder cancer case-control study. The objective of the epidemiologic research project is to identify a range of factors that have contributed to bladder cancer incidence in Michigan, with the focus on spatial and spatiotemporal patterns of exposure to naturally occurring arsenic in drinking water. Cases are recruited from the Michigan State Cancer Registry and diagnosed in the years 2000–2003. Controls are frequency matched to cases by age (± 5 years), race, and gender, and recruited using a random digit dialing procedure from an age-weighted list. To be eligible for inclusion in the study, participants must have lived in the eleven county study area for at least the past five years and had no prior history of cancer (with the exception of non-melanoma skin cancer). The goal is to enroll 1400 participants in total. This is an ongoing five year project and only some preliminary spatiotemporal datasets, visualization tools, and results are shown here. Conclusive results will not be available for a few more years, until data has been collected and analyzed for all 1400 participants. The STIS is being developed at BioMedware, in Ann Arbor Michigan with funding from the National Institutes of Environmental Health Sciences and the National Cancer Institute. In this paper STIS is used to visualize and analyze data from a bladder cancer case-control study but it can also be used for health/environment interactions or marketplace sales trends. More information about the STIS and a free 30 day download can be evaluated at .

## Results and discussion

Data from a case-control study of bladder cancer in south eastern Michigan was used to evaluate the efficacy of the STIS for documenting and visualizing space-time relationships between cases, controls and putative risk factors. Lifetime exposure to arsenic in drinking water (an element that has been associated with bladder cancer at high levels [12,13]) was reconstructed for each individual by incorporating spatiotemporal information about residential mobility (every address inhabited since birth), occupational history (every full time job since the age of 16), drinking water patterns, and concentration of arsenic in drinking water.

### Space time information system

The motivation for this system comes from the idea that the 'what and where' of conventional GIS needs to be extended to the 'what, where, and when' of reality and spatiotemporal modelling. Based on similar spatiotemporal approaches (e.g. [4], [18], [19]), objects are implemented using the space time model: {**object, space-time coordinate, attributes**} where **object **identifies the modelled entity (e.g. person X); **space-time coordinate **is a spatiotemporal location which may be a space-time point (e.g. latitude, longitude, altitude, date, movement model) or a space-time polygon (e.g. polygon centroid, polygon boundary, date, movement model); and **attributes **are observations on objects (e.g. income).

Within the **space time coordinate**, in addition to the well known descriptors (e.g. latitude, longitude), we also specify a movement model that defines how the object moves through space as a function of time. Among the simplest of movement models is an instantaneous displacement such that the object ceases to exist at one location and immediately reappears at another location. We use this simple model to describe residential histories.

**Morphing **describes how the shape of geographic features (such as lines and polygons) changes through time. Here an object is comprised of multiple vertices changing shape through time by the addition, deletion and movement of vertices. This is called network morphing (for lines) and polygon morphing (for polygons). Morphing can be gradual, in which case the change in the object's shape occurs over a defined time interval; or it can be abrupt. In our research we utilize this approach to model cadastral systems and the realignment of administrative and political boundaries. This allows us to track, for example, how municipal water districts change through time, and to then estimate arsenic exposure from drinking water for individuals on municipal water supplies.

**Attributes **are observations on variables describing the modelled entity and its environment (e.g. case/control identifier, population size, ethnicity, etc.) Our data model assumes observations occur at discrete times at which the attributes of an object are quantified. Attribute change models describe how the values of attributes change between observation times. The simplest attribute change model is a step function that updates an attribute's value when a new observation is made on that attribute. More complex change functions that obtain values from nearby locations are used to interpolate values through space and time for both categorical and continuous data [14]. These include techniques from the field of geostatistics that provide a probabilistic framework for space-time interpolation by building on the joint spatial and temporal dependence between observations [15]. In this research we use the step function approach to model, for example, change in arsenic concentration in potable water when an individual's water supply source is switched from one source of supply to another. We also use geostatistics to model how arsenic concentration in ground water changes spatially and as a function of geology (described in [16]).

### Study data

We reconstructed individual exposures by incorporating spatiotemporal data on residential mobility (where people have lived throughout their lives), water supplies (private well, city well water, or city surface water), and drinking water habits. Only locations in which the participants have lived or worked for longer than one year were collected and geocoded. Data about diet, smoking, and medical history were also collected by a phone interview or written questionnaire. A point file (where each point represents a participant) was then imported into the STIS along with associated database files containing attribute information such as address and primary source of drinking water. Table 1 is an example of the drinking water and residential mobility database. Even though information for only three participants is shown, seven different addresses and nine different sources of drinking water are represented. (Street addresses are not shown to protect participant's identity). Therefore, a change in address or primary source of water warrants a new row in the database.

**Table 1:** Part of database of participant addresses and water source information Information for four participants is shown. For each change in address or primary source of water a new row is entered in the database. Therefore there are 16 rows in this sample database.

**Year moved in**	**Year moved out**	**Sample ID**	**City**	**Primary Source of Water**
9/12/1935	1/1/1953	1	Swartz Creek	Private well
1/1/1956	1/1/1958	1	Swartz Creek	Private well, softener
1/1/1958	1/1/1963	1	Swartz Creek	Private well
1/1/1963	1/1/1974	1	Swartz Creek	Private well, reverse osmosis
1/1/1974	1/1/1990	1	Swartz Creek	new private well
1/1/1990	1/1/2002	1	Swartz Creek	Community Supply
1/1/2002	1/1/2004	1	Swartz Creek	Community Supply, softener
1/1/1976	1/1/1990	2	Livonia	Community Supply
1/1/1990	1/1/2004	2	Brighton	CS (township well and treatment plant)
1/1/1953	1/1/1961	3	Jackson	Community Supply
1/1/1961	1/1/1971	3	Jackson	Well (30 ft)
1/1/1971	1/1/1984	3	Michigan Centre	Private well
1/1/1984	1/1/1993	3	Vandercook Lake	Private well
1/1/1993	1/1/2004	3	Horton	Well (280 feet)
1/1/1943	1/1/1958	4	Ferndale	Community Supply
1/1/1982	1/1/2004	4	Waterford	Community Supply

Other point files were imported including present and historical data on industries and contaminated sites in the study area. A township map and water supply boundary map were imported as polygons. In addition to temporal changes in attributes such as township population, source of community's water supply, and number of people served, town boundaries and water supply boundaries changed with time. New towns were incorporated, community systems expanded their borders, and occasionally, communities were combined and town boundaries dissolved. All of these temporal changes were handled using attribute change models and morphing.

### Importing spatiotemporal datasets

We imported shapefiles describing the above data using the STIS data import facility that allows the variables to be time stamped. The user is prompted to import vector information into a new geography or an existing geography (if new information is to be added to an already existing geographic layer the latter will be chosen). The user must tell the system whether the data is (1) a *time slice *(similar to a collection of GIS static maps) where changes take place at specified times for all objects in the dataset, or (2) a *time series *where data varies asynchronously and objects move or change attributes at different times. For example Census data are time slice data – attributes remain constant for a decade (1980–1990) and then all attributes are updated with the next decade's census information (1990–2000). On the other hand, data associated with tracking residential histories are time series data, with household moves occurring at different times for each individual. The system imports data at temporal granularities varying from seconds to years; and the data may then be analyzed at these different time scales.

### Visualization procedures

Being able to visualize changes in boundaries and attribute values over time is an effective approach to better understanding and exploring data. Because time is a dimension of the data rather than an attribute all views of the data are easily animated. Analogous to a static GIS, attributes of data are visualized by specifying colour, shape, and size of graphical elements (e.g. symbols). However, in contrast to a GIS, the STIS easily facilitates visualization of changing polygon shapes and attribute values over time by animating maps, histograms, and tables simultaneously. Valuable information that might be lost in an atemporal GIS is captured and can become the focus of analysis in the STIS. There are four major visualization views – maps, graphs (histograms, scatter plots, box plots), tables, and time plots.

(1) The *map view *displays spatial data and the user interacts with the maps by zooming, panning, selecting, and querying. The added feature of the STIS is the animation toolbar. It is employed to show individuals changing place of residence through time; arsenic-emitting industries being founded, operating, and going out of business; municipal water supply districts growing and coalescing; and attribute values, such as arsenic concentrations, changing through time.

(2) In the STIS *histograms*, *scatter plots*, *and box plots *are also animated over time. An individual or group of individuals (e.g. cases vs. controls) may be selected at one point in time and the user can explore how that selection's values change through time. For example, we used this feature to explore how individual arsenic exposure changed over a participant's lifetime. We also used it to compare estimated arsenic burdens for the cases to those of the control population.

(3) *Table views *also are animated, as the given value of a variable (such as the arsenic concentration in a municipal water supply) will change through time. Tables thus show how data values change over time by updating a given objects value when it increases or decreases.

(4) The *time plot *graphs time on the x-axis and the value of a variable, such as estimated arsenic exposure, on the y-axis. Objects of interest, such as cases and controls, then map into this bivariate time plot to explore time dependencies in arsenic exposure. Unlike the other views, the time plot is not animated because it already shows the entire time range of the data on the x-axis.

A novel feature of the STIS is the ability to *time-link *visualization windows. Maps, statistical graphics, and tables may be time-linked so that all of the views are synchronized to the same point in time. Animating the time-linked windows then displays the views simultaneously changing through time. We use this feature to display the changing residential locations of the cases and controls along with the locations and emission volumes of arsenic-producing industries. All of this is done within the context of municipal water districts whose boundaries morph and whose arsenic concentrations are dynamic. While this map visualization is occurring we observe how the frequency distributions of modelled arsenic exposure are changing for the cases relative to that of the controls. Participants (cases or controls) are thus easily evaluated and compared to other participants in terms of their residential histories, and population-level characteristics, such as the mean and dispersion for arsenic exposure estimates, may be compared statistically as they evolve over time.

Statistical and Cartographic Brushing is employed to link together the views associated with a given dataset. This is made possible by using unique identifiers (such as the participant ID's of the cases and controls, or the names of the municipal water districts) to link together corresponding values on the maps and statistical graphics. Statistical brushing is used to select objects (such as the points on a scatter plot) and to then highlight the corresponding objects on maps and other statistical graphics. Cartographic brushing occurs when objects are selected on a map, and their corresponding values on the statistical graphics are highlighted. We used statistical brushing to select participants with high arsenic exposures, and to then identify their locations on maps of their residential histories. We use cartographic brushing to explore possible associations between proximity to arsenic emitting industries and the local densities of cases relative to the controls.

### Application of visualization procedures

**Figure 1:**
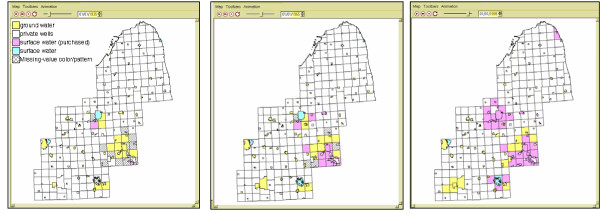
**Change in water supply systems over 50 years (1935, 1965, 1995) **Over the years many towns in Oakland County and Genesee County begin to purchase surface water (from Detroit).

We first investigate changes in the water supply systems (Figure 1). It is clear that over a 50 year interval (from 1935–1995) private well owners and some community ground water systems replaced their private wells or ground water systems with a purchased surface water system (hooking up to a larger system such as the Detroit Sewer and Water System). Visualizing this information over time is valuable as it shows areas that historically might have been associated with high arsenic levels. It also is used to help assign arsenic concentrations to previous residences. For some public ground or surface water systems historic arsenic concentrations have been recorded. For participants on such water supply systems we therefore can directly assign water source arsenic concentrations. Historic arsenic concentrations for well water supplies often are not available, and for these we interpolate arsenic concentration values using geostatistical procedures that account for values in nearby wells, spatial covariance in these values, and their dependency on predictors such as groundwater geology [16].

**Figure 2:**
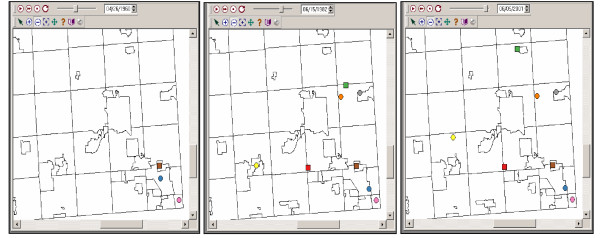
**Participant movement over 20 years **Cases (circles) and controls (squares) continue to move in, out, and around the study area. In 1960 there were two cases and one control. By 1982 four more cases and two more controls moved into the study area and in 2001 the same number of cases and controls remain in the area however one case and one control have moved addresses.

Visualizing the movement of bladder cancer cases and controls through time is crucial in our analysis of arsenic exposure and how it relates to the incidence of bladder cancer. Figure 2 presents participants at three different time points (1960, 1982, 2001). A case is represented by a circle and a control by a square. In 1960 there were two cases and one control. By 1982 four more cases and two more controls moved into the study area and in 2001 the same number of cases and controls remain in the area. Note that one case and one control have moved residences. The animated map thus informs us regarding the residential mobility of the cases and controls. Spatial and temporal subsets of these populations can then be selected and statistically analyzed and summarized using other visualization windows and statistical methods.

### Analysis of arsenic exposure

**Figure 3:**
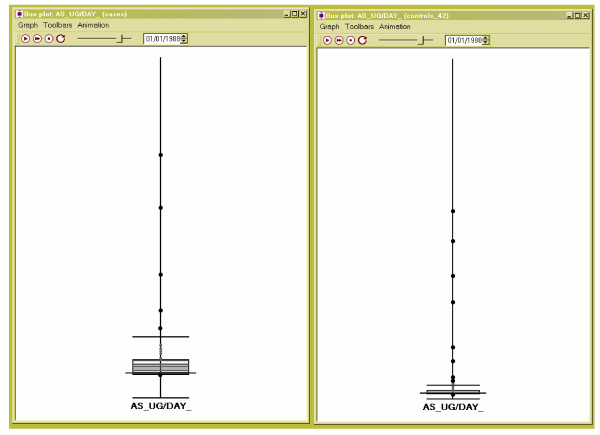
**Box plot of arsenic exposure in 1988 for cases (left) and controls (right) **The median is the black line that bisects the box. The upper and lower quartiles, the medians of the upper and lower halves of the data, are the edges of the black box. The "whiskers" on the box, the bars at the top and bottom, are 1.5X the interquartile range.

In this analysis we are interested in the temporal variability in arsenic exposure in cases versus controls as well as clusters of high arsenic values. Arsenic exposure was calculated by multiplying arsenic concentration (μg/L) by home consumption of water and beverages made with water (L/day) at each residence and for each change in water consumption. Data regarding water and beverage consumption was obtained via survey [17]. We utilize the box plot to look at means and interquartile ranges through time (Figure 3 for 1988). The windows are time linked and show cases (on the left) and controls (on the right). A more evenly distributed exposure to arsenic in the case subset is indicated by the large interquartile, and 1.5X interquartile range.

**Figure 4:**
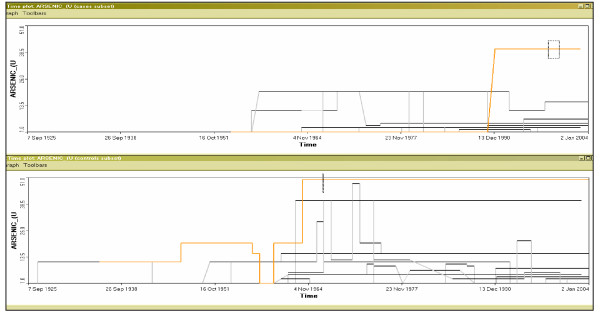
**Time graph of arsenic exposure in cases (top) and controls (bottom) **Notice the increase in arsenic for both sets after 1951. The increase in arsenic is much larger for controls and remains high for at least two individuals.

The time plot is another visualization method and provides information over the entire time range (Figure 4 x-axis equals time, y-axis represents arsenic exposure). This graph shows general trends in this preliminary dataset. In the early 1960's arsenic exposure was actually greater for controls (bottom graph) than for cases (upper graph). We also notice that the highest arsenic value (51 μg/L) occurred for a control in 1964 and lasted until the end of the study period. The highest value for a case (38 μg/L) occurred later in the study period (1990). All records are linked to the map view and an investigation of geographical clustering can occur in tandem with the temporal analysis of the time plot.

**Figure 5:**
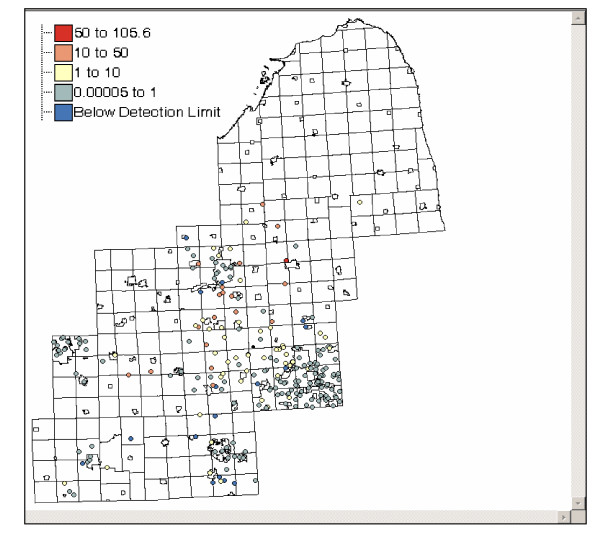
**Arsenic in drinking water (2003/2004) **Each point represents an arsenic value taken from the kitchen tap at the present residence of each participant.

**Figure 6:**
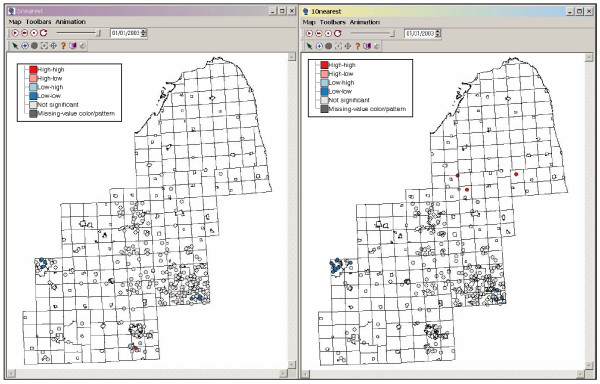
**Local Moran analysis at two spatial scales **Local Moran analysis with five nearest neighbours is on the left, and with ten nearest neighbours is on the right. Notice the appearance of the high-high cluster to the north, and the increase in size of the low-low cluster to the west as the size of the local neighbourhood is increased

In addition to the graphical analysis we employed statistical clustering methods to identify spatial clusters of homes with high arsenic concentrations in their water supplies. The Univariate Local Moran is a statistical method used to detect local spatial autocorrelation by decomposing Moran's I into contributions for each location. Here, each location refers to an arsenic value sampled at the home of each participant. Moran's I is a weighted correlation coefficient that is used to determine whether neighbouring areas are more similar than would be expected under the null hypothesis. In this study the local Moran statistic is used to detect where there are statistically significant clusters of high (or low) arsenic values in participants' drinking water. Data regarding arsenic in drinking water was collected at the kitchen tap of each participant from their present residence. Water samples were stored on ice, acidified with 0.2% trace metal grade nitric acid, and refrigerated until analysis. Water samples were subsequently analyzed for arsenic using an inductively coupled plasma mass spectrometer (ICP-MS, Argilent Technologies Model 7500 c) [17]. A map of arsenic values from participants' drinking water is shown in Figure 5. The Local Moran analysis was performed on this arsenic dataset resulting in a map of significant clusters (identifying areas as high-high clusters, low-low clusters, low-high outliers, high-low outliers, and areas not significant from background), and a local moran scatterplot. Figure 6 is the result of the local Moran analysis using spatial weights of five (left) and ten (right) nearest neighbours, with 999 randomizations, at the alpha level of 0.05. Generally the two maps look similar, and this is corroborated by similar Global Moran's I values of 0.126279 for five nearest neighbours and 0.129596 for ten nearest neighbours. However, there are differences that arise from analysing spatial pattern at two different local spatial scales. For example, in the northern region of the ten nearest neighbour map we find high-high values indicating high arsenic values surrounded by other high arsenic values. We also see an area of low-low values in the western part of the map, around Lansing. Households in these low-low locations are generally on community water supplies where arsenic values are kept below 50 μg/L to comply with Environmental Protection Agency standards. Conducting the Local Moran analysis at different neighbourhood sizes allows one to evaluate the sensitivity of clustering to different spatial scales.

## Conclusions

In this paper we presented a novel application of a space time information system to analyze some preliminary data in an ongoing case-control bladder cancer study. This approach is significant in that it not only visualizes the movement and attribute changes of spatial objects (including cases, controls, arsenic producing industries, and municipal water supplies) but also allows the user to compare values of these objects over time by time-linking windows. This ability to handle high temporal resolution data is enabling new approaches to exposure assessment. In the near future the STIS will be able to integrate exposure assessment models using an Application Programmers Interface (API). Users will have the flexibility to program specific models outside the software and then visualize their outcome in the STIS using the API. For less technically sophisticated users, a methods toolbar will be included, where common modelling algorithms will be made available using a simple calculator-type interface. Other plans for the software include importing and supporting raster files, exporting animated maps as movies (for presentations), visualizing geospatial lifelines [18,19] in a separate window once objects are selected, and adding spatiotemporal clustering statistics to the methods toolbar.

## Competing interests

Some authors are also affiliated with BioMedware a research company that also develops software for the exploratory spatial and temporal analysis of health and environmental data. With funding from the National Cancer Institute, GMJ, AMK, and GAA developed STIS, which is a commercial product of  Terraseer.
